# Supplementary material for: Analytical Technique Optimization on the Detection of β-cyclocitral in Microcystis Species
Source: Molecules. 2020 Feb 14;25(4):832. doi: 10.3390/molecules25040832 (PMC7070943; doi:10.3390/molecules25040832)
Supplement: Supplementary file 1 [file molecules-25-00832-s001.pdf]

molecules

MDPI

Supplementary Materials

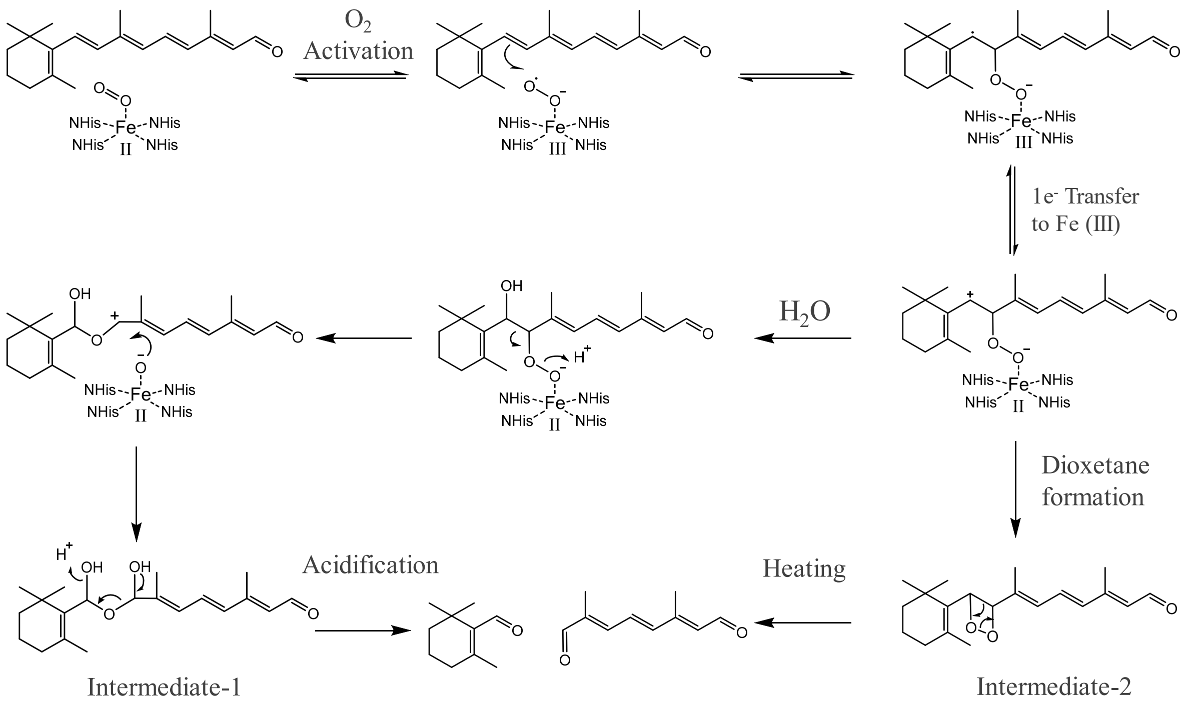

Scheme S1. Two plausible intermediates for the production of β-cyclocitral by CCD proposed by Hurrison and Bugg.
